# NNAlign: a platform to construct and evaluate artificial neural network models of receptor–ligand interactions

**DOI:** 10.1093/nar/gkx276

**Published:** 2017-04-12

**Authors:** Morten Nielsen, Massimo Andreatta

**Affiliations:** 1 Instituto de Investigaciones Biotecnológicas, Universidad Nacional de San Martín, 1650 San Martín, Argentina; 2 Department of Bio and Health Informatics, Technical University of Denmark, DK-2800 Lyngby, Denmark

## Abstract

Peptides are extensively used to characterize functional or (linear) structural aspects of receptor–ligand interactions in biological systems, e.g. SH2, SH3, PDZ peptide-recognition domains, the MHC membrane receptors and enzymes such as kinases and phosphatases. *NNAlign* is a method for the identification of such linear motifs in biological sequences. The algorithm aligns the amino acid or nucleotide sequences provided as training set, and generates a model of the sequence motif detected in the data. The webserver allows setting up cross-validation experiments to estimate the performance of the model, as well as evaluations on independent data. Many features of the training sequences can be encoded as input, and the network architecture is highly customizable. The results returned by the server include a graphical representation of the motif identified by the method, performance values and a downloadable model that can be applied to scan protein sequences for occurrence of the motif. While its performance for the characterization of peptide–MHC interactions is widely documented, we extended *NNAlign* to be applicable to other receptor–ligand systems as well. Version 2.0 supports alignments with insertions and deletions, encoding of receptor pseudo-sequences, and custom alphabets for the training sequences. The server is available at http://www.cbs.dtu.dk/services/NNAlign-2.0.

## INTRODUCTION

Sequence motifs are conserved, information-rich patterns in protein or nucleotide data. The specificity of sequence motifs governs a large number of signalling processes inside the living cell, including the active sites of enzymes, the binding preferences of membrane receptors (e.g. the MHC system) and DNA-binding proteins (e.g. transcription factors), as well as widespread modular peptide-binding domains like the SH2 and SH3 domains. Several computational methods have been developed to capture the sequence motifs at the base of these processes. The popular *MEME* suite (1) hosts a collection of tools for sequence motif analysis, enabling the discovery of ungapped (*MEME* (2)) and gapped (*GLAM2* (3)) motifs, as well as the detection of motif occurrences in a database (*FIMO* (4)). Other approaches to motif discovery include Gibbs sampling (5–7), Bayesian inference (8) and PWM optimization (9).

*NNAlign*, based on artificial neural networks (ANNs), was first developed in 2009 for the prediction of peptide–MHC class II binding affinity (10,11). Compared to most other approaches, ANNs can be trained on quantitative target data, and are therefore ideally suited to construct sequence-to-affinity models like the peptide–MHC system. Another advantage of ANNs is their ability to capture higher-order correlations, which can prove important to model positional correlations in receptor–ligand interactions. For these reasons, *NNAlign* has become the engine of some of the most successful tools for the prediction of peptide binding to MHC molecules, including *NetMHC* (12), *NetMHCpan* (13), *NetMHCII* and *NetMHCIIpan* (14), which have been shown to be the state-of-the-art by several independent benchmarks (see, e.g. (15–17)). However, the application of *NNAlign* is not limited to the MHC system, and we have employed it for example to generate models of protease cleavage (18).

Some of the recent improvements in *NNAlign* included the introduction of insertions and deletions (indels) in the sequence alignment, the encoding of the receptor pseudo-sequence, and the possibility to customize the alphabet of the training data. Indels remove the restriction of a binding core of fixed length, and in the context of the MHC class I system has allowed training models on peptides of all length with significantly improved performance (12,13). Encoding a receptor pseudo-sequence, i.e. the residues of the molecule in direct contact with the bound peptide, as input to a model has enabled the creation of ‘pan-specific’ predictors, with the ability to infer the binding specificities of uncharacterized receptors by similarity to known ones. This has proven extremely important for example in expanding the allele coverage of peptide–MHC class I and II affinity predictions (19,20). Finally, making the sequence alphabet customizable opens up numerous new opportunities beyond peptide data described by the standard 20-letter amino acids. They include the study of post-translation modifications (PTMs), as well as the identification of sequence motifs in DNA or RNA data sets. Published methods developed using *NNAlign* with these recent additions are listed in Table 1. These tools are all state-of-the-art, and have been generated in a fully automated manner using the flexibility of the parameter space of the *NNAlign* method.

**Table 1.** Published prediction methods based on NNAlign

NNAlign method	MHC class	Indels	Pseudo-sequence	PCC	AUC
NetMHC-3.4 ^a^	I	—	—	0.691	0.870
NetMHC-4.0 ^a^	I	X	—	**0.722**	**0.886**
NetMHCpan-2.8 ^b^	I	—	X	0.709	0.882
NetMHCpan-3.0 ^b^	I	X	X	**0.732**	**0.893**
NetMHCII-2.2 ^c^	II	—	—	0.664	0.838
NetMHCIIpan-3.0 ^c^	II	—	X	**0.735**	**0.871**

Features implemented in a given method are marked with an X. Methods with indels allow for insertions and deletions in the sequence alignment; methods without pseudo-sequence encoding are allele-specific, methods with pseudo-sequence encoding are pan-specific. Indels and receptor pseudo-sequence encoding are only available in NNAlign version 2.0.

^a^Performance values from (12).

^b^Performance values from (13).

^c^Performance values from (20). The best performing method within each class is highlighted in bold.

With the *NNAlign* server, we provide an open platform that allows users to create their own models of receptor–ligands interactions. The server takes as input a list of peptide sequences with target quantitative values (which could represent, for example, binding affinities, or a binary classification of positive and negative instances). The server will then construct an ensemble of artificial neural networks that aims to capture the binding motif underlying the sequence data. The submission page of the server offers a wide range of options, allowing the user to process the data in several ways and to customize many parameters of the neural network architecture. It also enables the user to evaluate the predictive performance of the method, by automatically creating data partitions and cross-validation set-ups. The results page returned by the server reports performance values of the cross-validation experiments, together with graphical representations of the sequence motifs that were identified in the data. It also allows downloading the neural network model created by the user, which can then be used again (through the web server) to generate predictions on any other additional peptide sequences. In this document we will describe the web interface, its output, and will provide some examples of usage.

## WEB INTERFACE

### Submission page

#### Training data

The essential input to the server is a file consisting of two columns: a list of peptides in the first column, and numerical target values in the second. Target values may be the measured binding affinity of a peptide to a receptor, an array spot intensity, or any other quantitative measure associated to the biological sequences. The program was designed with quantitative peptide data in mind (i.e. a spectrum of target numerical values) but also accepts binary data. In all cases it is important, for effective training of the neural networks, to include both positive and negative instances in the training data; if for a specific problem at hand only positive instances are available, one may for example generate artificial negatives and assign them a target value of zero. The submission page includes examples of training data, and a button to automatically upload sample data.

#### Options and parameters

Several parameters can be specified to customize the models built by *NNAlign*. Basic options include a textual identifier for the run, and the width of the alignment window. The latter can be specified as an interval of values, in which case the server will suggest the optimal motif length as part of the output. The architecture of the network is highly customisable (e.g. the size of the hidden layer, the number of initial random weight configurations) and allows encoding multiple aspects of the input data. An important feature of the server is the possibility to automatically set up cross-validation experiments, with partitions generated by several alternative algorithms. Cross-validation allows estimating the predictive performance of the models generated on your data, before it is applied on independent sequence sets. Important new features introduced in version 2.0 include insertions and deletions (indels) in the sequence alignment (implemented as described in (12)), and the option to specify custom alphabets for the input data (demonstrated further in this document as a case study on DNA sequences). Note that when the training data are in an alphabet different from the standard 20-letter amino acid code, Blosum substitution scores cannot be applied to encode the sequence data and the representation is made using sparse encoding (18). If the training examples are associated with multiple receptors of known sequence, the user can also specify the receptor names as a third column in the training file, and then upload the receptor pseudo-sequences through the dedicated option. This mode enables the generation of ‘pan-specific’ models (21,22). A comprehensive description of the options, including usage guidelines, can be accessed by clicking on the ‘Instructions’ tab of the *NNAlign* submission page.

#### Model upload

When a job is completed, the resulting model can be saved to local disk. Models can then be uploaded to the main *NNAlign* submission page by checking ‘Upload a model’ in the top drop-down window. Models can be applied to generate predictions on external evaluation data in peptide or Fasta format (see below).

#### Evaluation data

The model generated on the training data can be optionally applied on an independent evaluation data set. The evaluation set can be either a list of peptides, or full protein sequences in Fasta file. Fasta submissions are converted into peptides by digesting them into overlapping peptides of length specified with the corresponding option.

### Output page

The output page details the outcome of the neural network training both in terms of identified sequence motif and in terms of model performance. Predictive performance is estimated in cross-validation on the training set, and displayed in terms of root mean squared error (RMSE), Pearson's correlation coefficient (PCC) and Spearman's rank correlation (SRC). For a visual representation of the correlation between target and predicted values, a scatterplot is automatically generated and can be accessed through a link on the results page. The binding motif defined by the sequence alignment is visualized directly on the results page with a sequence logo generated with the program Seq2Logo (23), and is also available in the form of position specific scoring matrices (PSSM). Copying and pasting the log-odds matrix into the Seq2Logo submission form allows further customization of the motif logo, including alternative color codes and different types of logos. The model generated by the server run can be downloaded and saved to disk. It contains all the model parameters and network weights necessary to run the program and generate predictions on new sequences. A link for bulk download at the bottom of the results page generates a compressed folder containing all the results, including plots, sequence logos and alignment files.

## EVALUATION AND EXAMPLES

### Determining the binding specificity of HLA-DRB1*03:01

**Figure 1.**
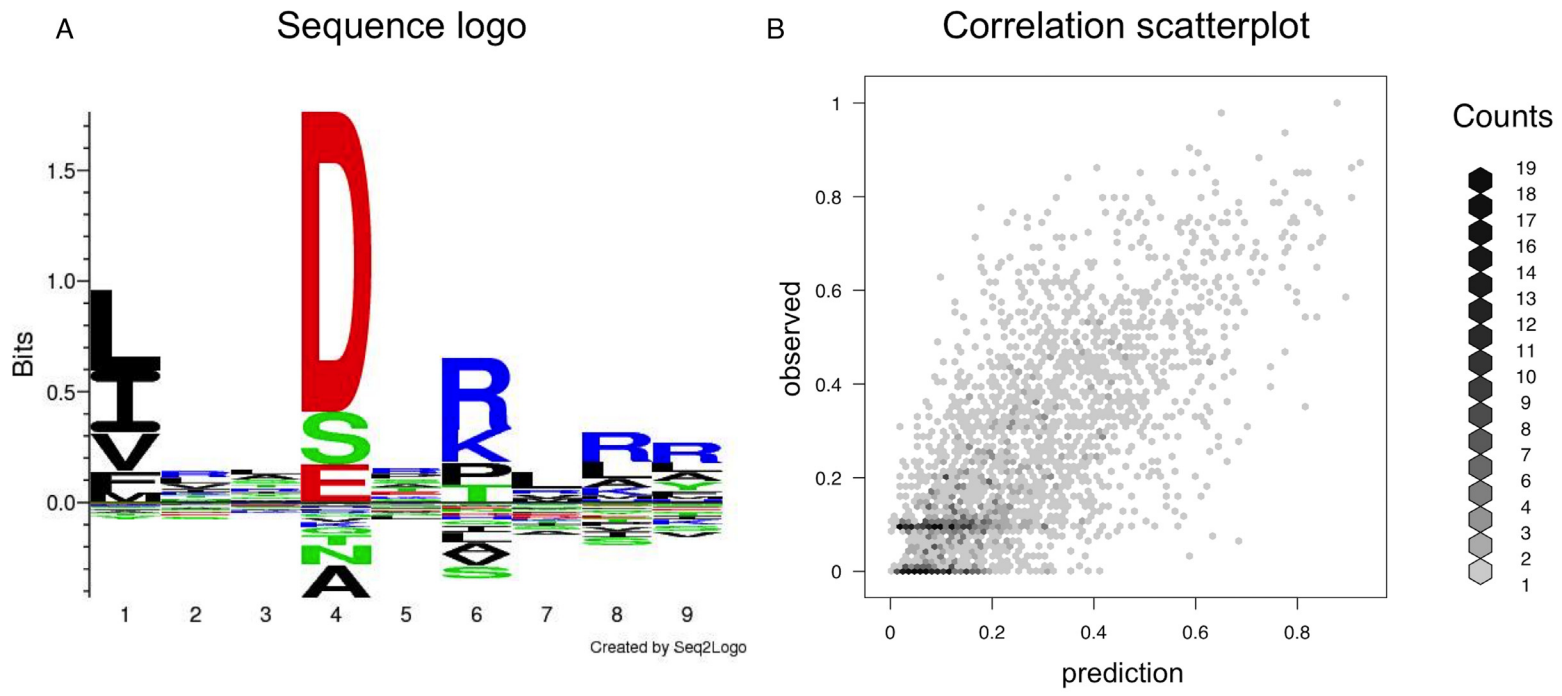
(**A**) Sequence motif identified by NNAlign for the binding specificity of HLA-DRB1*03:01 showing distinct amino acid preferences at the anchor positions P1, P4 and P6. (**B**) Correlation between the target and predicted log-affinities of the training data, calculated in cross-validation; in this example PCC = 0.721 and SRC = 0.702. Both plots are automatically generated by the NNAlign server and displayed as part of the output.

As an example application of the *NNAlign* server, we show how one can effectively determine the binding specificity of a HLA molecule with just a few minutes of work. We extracted a list of 2,505 binding IC50 affinity measurements to HLA-DRB1*03:01 from the Immune Epitope Database (IEDB) (24), rescaled between 0 and 1 using the relationship 1-log(IC50)/log(50,000) (25). We upload these peptides as training data, specify an expected peptide length for encoding of 15, and otherwise leave all other parameters to default values. Within a couple of minutes the server responds with the output page, which contains all the details about the model being generated. The sequence logo (Figure 1A) shows that the networks have identified distinct specificities at positions P1, P4 and P6, which correspond to known anchor positions for many MHC class II molecules (26). The performance measures estimated in cross-validation are RMSE = 0.165, PCC = 0.721 and SRC = 0.702. As part of the output, a scatterplot of the target versus predicted values (Figure 1B) gives a visual interpretation of the correlation between observations and predictions. By saving the model to disk, we can subsequently return to the *NNAlign* submission page, and apply the model to scan proteins or peptide data sets to identify novel potential HLA-DRB1*03:01 binding peptides.

### Training a multi-receptor model of peptide–MHC binding affinity

Next, we illustrate how the *NNAlign* method can be applied to train multi-receptor models. We generated a sample dataset containing binding data for multiple receptors by extracting from the IEDB 1,500 peptide-MHC affinity measurements for 8–11mer peptides associated to three HLA class I molecules (500 data points each for HLA-A*01:01, HLA-B*07:02, HLA-B*08:01). In order to train a multi-receptor model, the input data must consist of three columns: 1) the peptide sequence; 2) the target value; 3) the name of the receptor associated to the peptide-target pair. The ‘Load receptor pseudo-sequence’ option provides the method with the aligned pseudo-sequences that characterize the three MHC receptors; in this case, we used the string of 34 polymorphic amino acids of the MHC directly in contact with the peptide, as previously described for the *NetMHCpan* method (21). We specified a motif length of 9 amino acids, and allowed up to two deletions and one insertion. All other options were left at default value. As a comparison, we generated a model with identical parameters, but omitting the pseudo-sequence encoding.

**Figure 2.**
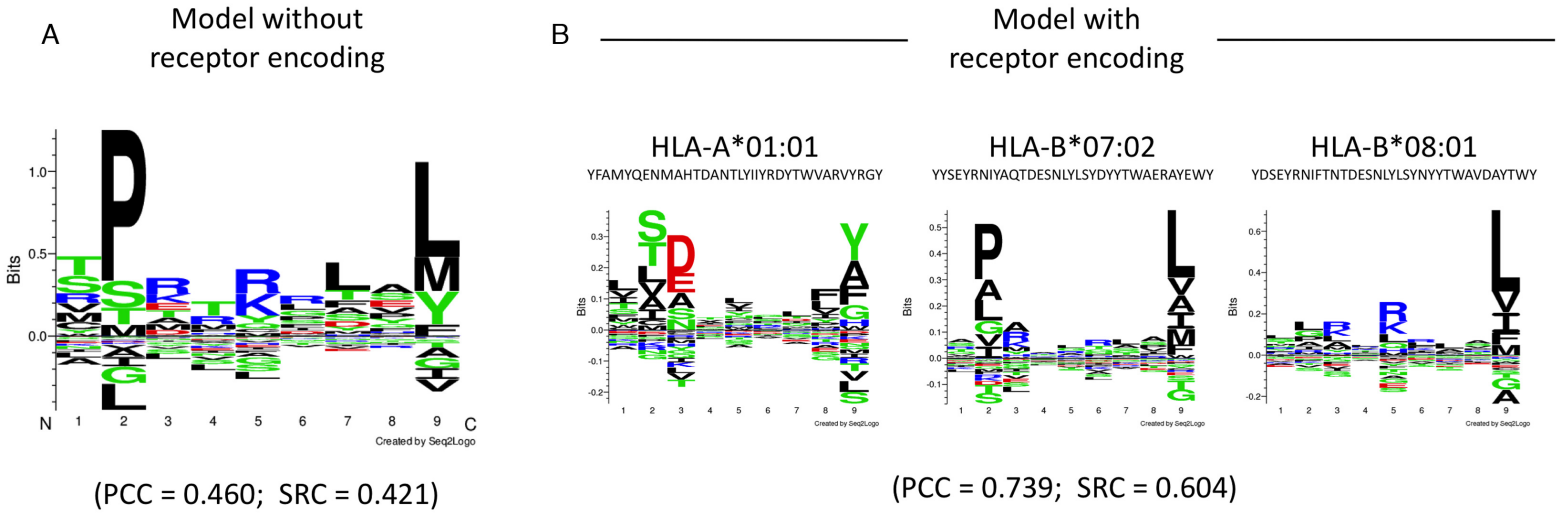
Sequence motifs identified in a mixture of HLA class I binding data. (**A**) On unlabeled data, NNAlign generates a motif that is an average of the three specificities contained in the training data. (**B**) If training data points are labelled with the pseudo-sequence of their receptor, the NNAlign model can learn the different specificities contained in the data. Receptor pseudo-sequences are indicated under their respective HLA receptor name.

The *NNAlign* model trained without the receptor sequence encoding returned a cross-validated performance of PCC = 0.460 (SRC = 0.421), and learned a motif that is a mixture of the binding preferences of the three molecules (Figure 2A). In contrast, the multi-receptor model (PCC = 0.739, SRC = 0.604) could successfully map the training data points to their respective pseudo-sequences, and learned binding motifs specific to the three different receptors (Figure 2B). Note that sequence logos are not automatically generated by the server in multi-receptor mode, but can be derived by submitting a large evaluation set of natural random peptides and selecting the top 1% scoring sequences for each receptor. On a larger scale, this multi-receptor approach was used to develop methods like *NetMHCpan* and *NetMHCIIpan* (see Table 1), where several hundred receptors were encoded into a single neural networks model.

### Changing the alphabet: transcription factor–DNA binding motifs

To illustrate how *NNAlign* can readily be applied to custom alphabets other than the standard 20 amino acids one-letter codes, we applied the method on a set of DNA sequences to characterize transcription factor (TF) binding sites. The training data for the DREAM5 TF–DNA Motif Recognition Challenge (27) consists of protein binding microarray (PBM) probe intensity signals for 66 murine TFs, using two different array types with ∼40 000 spots per array. Probe intensities were provided only for 33 TFs from one array type and the remaining 33 TFs from the other array type. The goal of the competition was to predict the unknown probe intensities for all 66 TFs.

**Figure 3.**
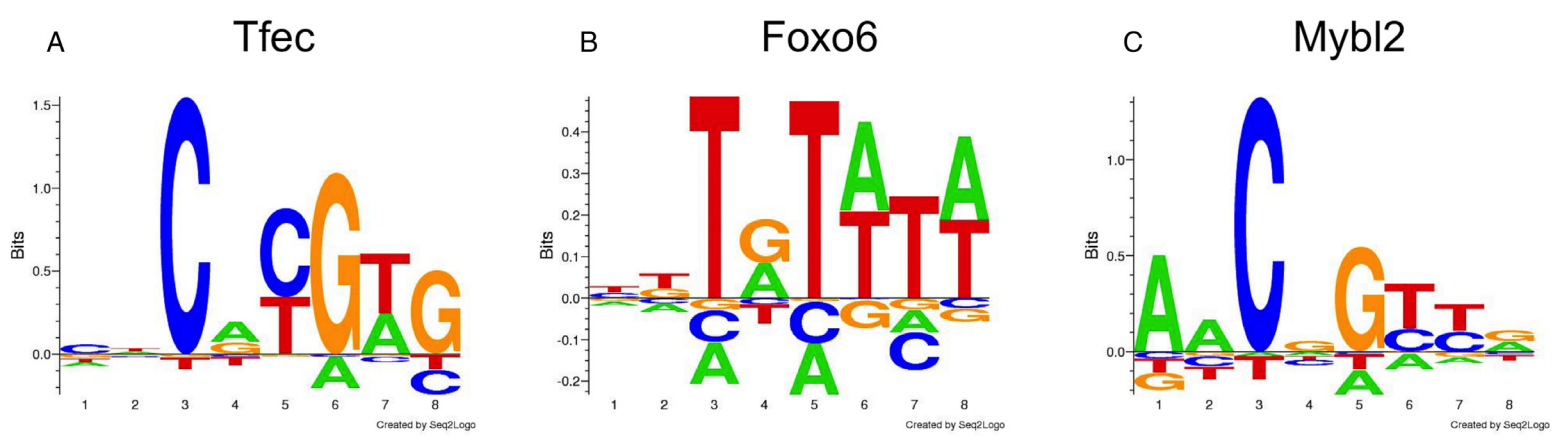
Sequence motifs identified by NNAlign for the three transcription factors Tfec (**A**), Foxo6 (**B**) and Mybl2 (**C**), derived from the PBM data of the DREAM5 TF–DNA Motif Recognition Challenge.

We generated *NNAlign* models on the training data of the challenge, specifying the custom alphabet ‘ACGT’, setting a motif length of 8 nucleotides, log-rescaling of the data and using two hidden layer architectures composed of either 15 or 50 neurons. The average PCC over the 66 TFs for the prediction of the blind set of probe intensity was of 0.612. The best performing method in the DREAM5 challenge obtained a PCC of 0.696. Although the performance of *NNAlign* in this situation is lower than that obtained by state-of-the art algorithms specifically developed for the task of TF identification, it is important to keep in mind that this performance was achieved with very limited effort and optimization of model parameters. The motifs identified by *NNAlign* are in general agreement with published TF-DNA binding preferences, for example those deposited in the CIS-BP database (28). In Figure 3 are shown the sequence logos generated by NNAlign for three transcription factors in the challenge: Tfec (TF26), Foxo6 (TF3) and Mybl2 (TF45).

## DISCUSSION

Modern high-throughput methods such as DNA and peptide microarrays, ChIPseq, and tandem mass-spectrometry have made it possible to study receptor–ligand interactions with parallel measurements of thousands to hundreds of thousands of probes in a single experiment. Many computational methods have been developed to analyse this kind of data for specific biological systems, as exemplified by the high number of participants to competitions such as the DREAM5 TF–DNA Motif Recognition Challenge (27) and the Machine Learning competition in Immunology (MLI) for peptide–MHC binding predictions (29). Methods than can be applied to different kinds of biological sequences, and in particular than can take advantage of the quantitative nature of certain assays, are much more rare. *NNAlign*, a motif discovery method based on neural networks, has a well-documented and robust performance for peptide–MHC binding prediction. Several of the recent improvements to the method, including the ability to generated gapped sequence alignments and the extension of the input sequence format to any custom alphabet, have greatly increased the generality of the method and its applicability to different biological systems. With the *NNAlign* server, we provide an online platform that allows users to create their own models of receptor–ligand interactions, and apply these models to detect motif occurrences in proteins or genes.

Sequence logos (30) are a compact, intuitive representation of a sequence motif and have been widely used to depict the nucleotide or amino acid preference of a receptor specificity. However, higher information content and a ‘sharp’ motif are not necessarily equivalent to a better representation of the preference of a receptor; it has been shown that the best predictors in many cases have more degenerate motifs with lower information content compared to methods that focus too much on the consensus sequence (27). This is especially true for a method like *NNAlign*: artificial neural networks can learn non-linearity in the data, and a linear representation such as a sequence logo will only be an approximation of the motif(s) learned by the networks. Although tremendously useful and intuitive, sequence logos should always be considered together with the performance values of the model that produced them. In this sense, *NNAlign* is unique in that it provides both a visual representation of the motif and an estimate of predicted performance of the model, as well as a usable model that can be applied to discover occurrences of the motif in biological sequences.

We have here illustrated how the *NNAlign* method can be readily applied to effectively and accurately characterize motifs in different types of biological protein and DNA/RNA ligand data sets. Thanks to its simple interface, the flexibility to handle a large variety of data set types, and its documented robust performance, we believe *NNAlign* could become an important tool to guide the analysis and interpretation of large-scale peptide data sets by users with limited bioinformatics expertise.
